# Mold-free self-assembled scalable microlens arrays with ultrasmooth surface and record-high resolution

**DOI:** 10.1038/s41377-023-01174-7

**Published:** 2023-06-07

**Authors:** Zhihao Liu, Guangwei Hu, Huapeng Ye, Miaoyang Wei, Zhenghao Guo, Kexu Chen, Chen Liu, Biao Tang, Guofu Zhou

**Affiliations:** 1 Guangdong Provincial Key Laboratory of Optical Information Materials and Technology & Institute of Electronic Paper Displays, South China Academy of Advanced Optoelectronics, South China Normal University, Guangzhou, 510006 China; 2 National Center for International Research on Green Optoelectronics, South China Normal University, Guangzhou, 510006 China; 3 School of Electrical and Electronic Engineering, 50 Nanyang Avenue, Nanyang Technological University, Singapore, 639798 Singapore; 4 Shenzhen Guohua Optoelectronics Tech. Co. Ltd, Shenzhen, 518110 China

**Keywords:** Optics and photonics, Photonic devices

## Abstract

Microlens arrays (MLAs) based on the selective wetting have opened new avenues for developing compact and miniaturized imaging and display techniques with ultrahigh resolution beyond the traditional bulky and volumetric optics. However, the selective wetting lenses explored so far have been constrained by the lack of precisely defined pattern for highly controllable wettability contrast, thus limiting the available droplet curvature and numerical aperture, which is a major challenge towards the practical high-performance MLAs. Here we report a mold-free and self-assembly approach of mass-production of scalable MLAs, which can also have ultrasmooth surface, ultrahigh resolution, and the large tuning range of the curvatures. The selective surface modification based on tunable oxygen plasma can facilitate the precise pattern with adjusted chemical contrast, thus creating large-scale microdroplets array with controlled curvature. The numerical aperture of the MLAs can be up to 0.26 and precisely tuned by adjusting the modification intensity or the droplet dose. The fabricated MLAs have high-quality surface with subnanometer roughness and allow for record-high resolution imaging up to equivalently 10,328 ppi, as we demonstrated. This study shows a cost-effective roadmap for mass-production of high-performance MLAs, which may find applications in the rapid proliferating integral imaging industry and high-resolution display.

## Introduction

Microlens arrays (MLAs) can support the large field of view angle and infinite depth of field in principle, and have become the essential optical component in vast applications, ranging from optical sensors^1^, Lidar systems^2^, light-field cameras^3,4^, optical microscopes^5^, high throughput maskless lithography^6^, to three-dimensional (3D) imaging and displays^7,8^. With the advances in modern foundry fabrication techniques, MLAs featuring high resolution and low aberration offer a fantastic paradigm for developing miniaturized and high-resolution 3D photography^1^, integral imaging^4,7,8^ and autostereoscopic display^9,10^, far beyond the reach of traditional optics^11,12^. However, the realization of MLAs with controlled focal length, high degree of perfection and high fill-factor, which critically determines the resolution and performance of the associated optical systems, remains challenging or costly, thus hindering the rapid development of high-resolution 3D photography and displays.

So far, MLAs with high fill-factor have been mostly produced through inkjet printing^13^, laser direct writing^14,15^, screen printing^16^, lithography^17^, photopolymerization^18^, hot melt reflow^19^, nanoimprinting^20^, and chemical vapor deposition^21^. These preparation methods can be predominantly categorized into direct method and indirect method^22^. The indirect method relies on mold and replicates the microlens based on molding or injection molding, where the geometry of microlens is determined by the mold^23^. A major challenge of this method lies in the complicated and costly fabrication process of the mold or patterned photomask. Moreover, post-processing, such as peeling off and transfer, may damage the surface profile^24,25^, thus leading to defects in surface profile, poor uniformity and varied curvature.

This challenge can be mitigated by using the direct method, which depends on the surface tension and function in case of thermoplastic or liquid material^26^. On the one hand, the surface tension domination in fabrication enables surface topography with roughness down to 1 nm in case of microlens^27–29^. On the other hand, the direct method allow large-scale micron droplet array by direct coating, thus enabling the mass production of MLAs by using self-assembly^16^. However, since their geometry is determined by control parameters such as temperature^19^, wettability^16^, liquid dose and processing time^27,30^, the precise control of curvature and focal length remains difficult. In this case, mould-assisted techniques are also usually introduced in the direct method to set a boundary constraint for the thermoplastic or liquid material in the self-assembly process, while the roughness is controlled by balancing the surface tension and the gravity^28,29^. Nevertheless, this still requires the sophisticated lithography to fabricate the mold. So far, a real mold-free approach to manufacture high-quality MLAs with ultrahigh resolution, without relying on complex machining or expensive infrastructure, is highly demanded.

Herein, we report a cost-effective approach to produce MLA with scalability by selective wetting. We use oxygen plasma to modify the hydrophobic interface to selectively create well-defined hydrophilic area, which provides strong restriction for the curable liquid instead of using conventional mold. The numerical aperture of the MLA can be up to 0.26 due to the strong chemical bonding force arising from the tremendous wettability contrast and precisely controlled by adjusting the modification intensity or the droplet dose. The obtained MLA can have high-quality surface with subnanometer roughness (0.34 nm) and allow for record-high resolution imaging up to equivalently 10,328 ppi (see Table 1). This preparation technique based on selective wetting may be extended to produce MLA in batch with low cost by adopting blade coating. This study demonstrates an effective roadmap to produce MLA, and may find applications in integral imaging.

**Table 1:** Summary of previously reported microlenses array

Reference	Minimum diameter (μm)	Height (μm)	Gap (μm)	NA	Resolution (μm)	ppi	Roughness (nm)
Feng et al.^29^	33	1.9	~20	NA	~100	~254	0.98
Xu et al.^27^	200	~12	~50	~0.16	62.5	406	NA
Bae et al.^17^	40	9.5	25	0.21	12.8	1984	NA
Zhou et al.^16^	200	38	33	0.06	NA	NA	NA
Kim et al.^30^	53	~12	100	~0.3	NA	NA	NA
This work	10	5.2	10	0.26	2.46	10,328	0.34

## Results

### Mechanism and fabrication of MLAs

**Fig. 1:**
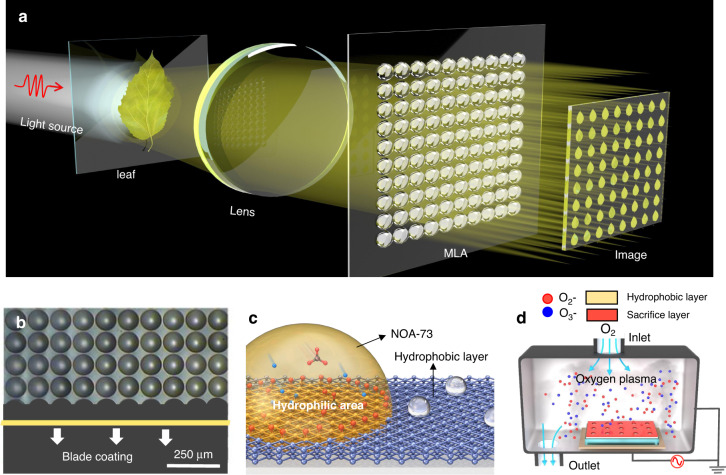
Schematic illustration of the MLA for integral imaging by selective wetting the hydrophobic interface with oxygen plasma. **a** Schematic diagram of the MLA for integral imaging. **b** Produce of MLA based on blade coating technique and curable liquid, where microlenses are tied to the well-confined surface. **c** The mechanism of selective wetting. The curable liquid NOA-73 is confined in the hydrophilic area. **d** Oxygen plasma modification process based on reactive ion etching. Oxygen plasma is adopted to modify the hydrophobic interface so as to selectively create a well-defined hydrophilic area, which provides strong restriction for the curable liquid by introducing chemical bonding force

Figure 1a depicts the schematic illustration of adopting MLAs for integral imaging realized by selectively wetting the hydrophobic interface with oxygen plasma. The core idea is to adopt two MLAs to capture the target and then reproduce the light field so as to realize an autostereoscopic 3D display. Taking a leaf for example, arrayed images of the object are captured by the first MLA, processed by the display system and then projected by the second MLA before it enters human eyes. Hence, the properties of the MLA, such as uniformity, surface roughness and resolution, play a vital role in determining the performance of the display system. Figure 1b illustrates the image of the experimentally fabricated MLA with diameter of 100 μm. We manufactured the MLAs by using blade coating method, where the curable liquid NOA-73 sticks to the selective wetting surface (hydrophilic area, as indicated in Fig. 1c) and forms into monoconvex lens, due to the surface tension and the chemical bonding force between the interfaces.

Instead of utilizing conventional mold to provide physical boundary for the curable liquid^23^, both hydrophilic and hydrophobic surfaces are created to provide strong restriction for the curable liquid in this study. As shown in Fig. 1d, oxygen plasma is adopted to modify the hydrophobic interface and selectively create a well-defined hydrophilic area. The sample with sacrificial layer is put into the chamber of the reactive ion etching machine. The ionized oxygen plasma impinges upon the hydrophobic surface in the chamber to induce its transition to hydrophilic surface. In our experiment, an amorphous fluoropolymer Hyflon is adopted as hydrophobic material on the glass substrate, because they show near-zero extinction coefficient and approximately constant refractive index (with ±0.005 variance during wavelength of 400 nm~660 nm) in the visible and near infrared range (see the ellipsometer measurement results in Fig. S1), suggesting the negligible material loss and weak dispersion. To understand the microscopic change in surface chemical properties before and after O_2_-plasma modification, X-ray photoelectron spectroscopy (XPS) is employed (see Fig. S2 for more details in supporting information). This suggests the enhancement of surface activity induced by the surface defluorination, and thus the surface hydrophilicity. As the binding force caused by chemical bonds on the solid (Hyflon)-liquid (NOA-73) interface is much stronger than traditional solid-liquid binding by van der Waals force^31^, more binding is produced in the locally modified region, thus supporting the formation of selective-wetting-dominated microdroplet array during the blade coating process. Hence, compared with MLAs relying on conventional mold^19,32^, the hydrophilic layer may support MLA with larger height, implying that this method may lead to MLAs with sharper curvature and higher numerical aperture (NA). Moreover, the chemical restriction method helps abandon the mold relying on high-precision nanofabrication, and thus becomes a rather cost-effective method to fabricate the MLAs as blade-coating or slit-coating can be applied.

**Fig. 2:**
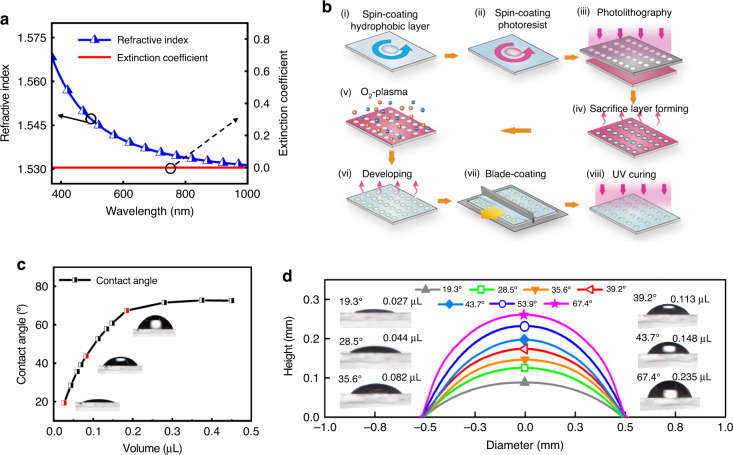
Fabrication procedule and surface profile control of microlens. **a** Refractive index and extinction coefficient of NOA-73. **b** The manufacturing process of MLA based on selective wetting. **c** Relationship between the contact angle of lens and droplet volume. **d** Profile of lens cross section and the contact angle image of lens morphology regulated by droplet volume in 1 mm modified region

We first consider curable liquid NOA-73 as fundamental material to form the lens. Figure 2a shows the weak material dispersion as its refractive index is approximately constant as 1.54 in the visible and the negligible extinction coefficient, making it ideal for broadband and high-efficiency optical lens. The curable NOA-73 coated onto an array of well-confined circular hydrophilic area will show self-assembly and morph into MLAs to the size of hydrophilic areas (with a diameter of 1 mm shown in Fig. 2). We want to highlight that previous methods in this step rely on a lens-shaped mold, posing a stringent requirement on the surface quality and machining accuracy of the mold for high-quality MLAs, let alone the additional cost^32^. In striking contrast, our study tailors the surface tension and chemical bonds between the droplet and modified areas to provide strong viscous force to restrict the morphology of the MLAs, opening a path toward the mold-free and cost-effective fabrication of large-area high-quality MLAs.

Figure 2c shows the relationship between the volume of NOA-73 and contact angle of the MLA using Stylus Profilometer (Dektak XT, Bruker) and Optical Goniometer (JC-2000D1, Powereach). When increasing the volume, the contact angle changes from 18° to 76° (Fig. 2c). To test the 3D surface profiles of the MLAs, we use Optical Microscope (OM) (CSUC-200C, Hours) and 3D Surface Profilometer (DCM8, Leica). The 2D images of the cross sections of six lenses are displayed in Fig. 2d. It can be observed that the lenses are well-confined inside the region where the Hyflon is modified. As NOA-73 increases from 0.027 μL to 0.235 μL, the height of the resulting MLA morphs from 0.08 mm to 0.25 mm accordingly (see the inset in Fig. 2d) accompanied by the increase of contact angle up to 76°. Interestingly, we note that the diameter of the MLAs remains the same in this process, suggesting the configuration of the curvature and NA of the MLA by controlling the dose of NOA-73. Ideally, single microlens in the MLA can be considered as a spherical cap composing of NOA-73. If the circular-shaped hydrophilic area has a diameter *d* and the dose of the liquid is assumed as *V*, then we have *V* = *πH(d*^*2*^*/8* + *H*^*2*^*/6)*, where *H* is the height of the microlens. Hence, the curvature of the MLA can be configured according to demand by tuning the dose of the NOA-73. Moreover, we find in the experiment that the curvature and NA of the MLA can also be scaled by tuning the time and the dose of the oxygen plasma during modifying the hydrophobic interface. Although the contact angle is expected to saturate after certain threshold volume (0.25 μL in our case) due to the crash of liquid by gravity, the further engineering of hydrodynamic properties of fundamental materials may enable a larger contact angle, which would be interesting for future study.

**Fig. 3:**
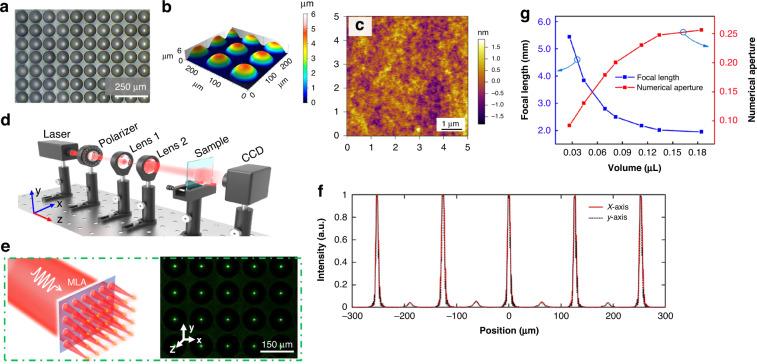
Optical characterization of the experimentally fabricated MLA. **a** The morphology image of the MLA with aperture of 100 μm. **b** Three-dimensional morphology of single microlens in the array. **c** The surface roughness of the MLA tested by atomic force microscope. **d** Schematic illustration of the experimental setup to characterize the samples. **e** The focal spots experimentally recorded in the focal plane. **f** The experimentally recorded electric-field intensity distributions along the x- and y-direction at the focal plane. **g** The relationship between the liquid volume and the focal length, and NA

Figure 3a, b shows the OM image and the 3D surface profile of a MLA with periodicity of 200 μm, respectively. The height of the MLA is ~6 μm, while the diameter is measured to be 100 μm, which matches with the design. These results indicate that the MLA has an excellent uniformity. It can also be noticed that no liquid overflows into the hydrophobic region, rendering the MLA neat. To test the surface roughness of the MLA, we use the atomic force microscope here. Figure 3c depicts the surface roughness of a microlens randomly selected from Fig. 3a, where an average roughness of 0.34 nm is estimated. The polishing procedure which is usually employed in traditional fabrication techniques is no longer necessary here^33^. MLAs with such smooth surfaces are promising for those applications where the highly uniform MLAs with even dense arrays and ultrasmooth surface are demanded.

### Optical characterization of MLAs

We now present the experimental characterization of MLAs (see setup in Fig. 3d). Continuous-wave semiconductor laser with working wavelength of 532 nm with linear polarization is used. The laser beam is focused by the MLA, and the diffraction field is recorded by the current charged device (CCD). We first test the focusing properties of MLA with 100 μm aperture size. The diffraction pattern recorded by the CCD is displayed in Fig. 3e, where a uniform array of spots can be clearly seen. We calculate the quadratic variance of the light intensity of the foci, and the calculated variance of 10 × 10 microlenses is about 0.09, which indicates that the MLA has high uniformity. Furthermore, the light field intensity distribution along the transverse directions, namely *x* and *y* axis, are also measured and plotted in Fig. 3f, which helps to verify that the MLAs have an excellent lensing functionality. At the focal plane, the light field intensity distributions along *x* (black dahsed line) and *y* (red line) axis are largely overlapped, as shown in Fig. 3f. These results strongly corroborate the rotational symmetry and hence high surface quality of our sample.

As discussed, the height and contact angle of the MLAs can be tuned by controlling the liquid dose. If the region of the modified surface is fixed, the contact angle will reach a maximum. This limit cannot be surpassed even though the liquid volume is increased. Hence, in order to assess how the liquid dose affects the MLAs, the relationship between the liquid volume, focal length and the numerical aperture are fully studied here. As depicted in Fig. 3g, the focal length of the MLA (blue line in Fig. 3g) decreases quickly from ~5.5 mm to 2 mm when the liquid volume increases to 0.12 μL. This phenomenon can be attributed to the enlargement of the curvature of the MLA. The focusing ability of the MLA becomes stronger when the liquid volume increases. However, it can also be noticed that the focal length increases slowly when the volume is larger than 0.12 μL. In this case, the height of the MLA is raising slowly, because the gravity becomes dominant and the surface tension is not strong enough to against the increasing gravity. Consequently, the NA of the MLA also has a maximum due to the finite curvature even though the liquid volume increases. In this study, the NA of the MLA is calculated with equation NA = D/ 2*f*, where D is the aperture size and f is the focal length. The measured NA as a function of the liquid volume is plotted in Fig. 3g (red line). It can be seen the maximum NA is ~0.26 when the aperture size is fixed to 1 mm. The accuracy to obtain MLA with random NA depends on the accuracy of the control of the liquid volume, which is rather difficult to precisely control, especially in case of using blade coating. Alternatively, this obstacle can be overcome by controlling the intensity of the plasma in the surface modification, which determines the chemical bonding force and thus the height of the MLA. Therefore, customized NA may be realized based on this technique, rendering it promising for the applications in the proliferated 3D display industry^7–10^.

**Fig. 4:**
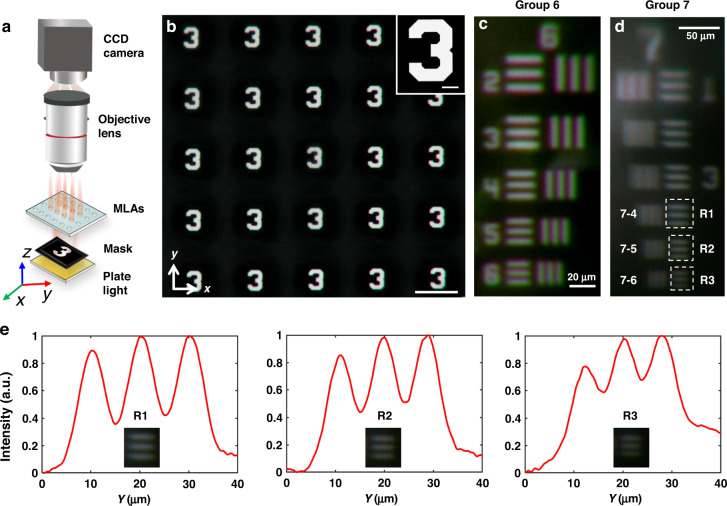
Optical characterization of the resolution of the experimentally produced MLA with diameter of 100 μm. **a** Schematic diagram of the experimental setup to test the resolution of the MLA. **b** The captured image of the sample after the MLA. **c**, **d** The captured images of the samples on the resolution plate (1951 USAF, Thorlab). **e** Analysis of the resolution of the MLA. The light field intensity distributions along the y-direction are corresponding to the recorded images (R1-R3) in Fig. 4d, respectively

The resolution of the MLA and the probability of using it in high-resolution imaging are experimentally explored and discussed in this study. For this purpose, we perform imaging experiment under the plate white light emitting diode (LED) illumination of MLA with diameter of 100 μm (Fig. 4a). An Arabic letter ‘3’ in the standard resolution test target (1951 USAF, Thorlab) with a linewidth of 75 μm is employed as an imaging object, as shown in the right top inset of Fig. 4b. Assisted by our MLA with focal length of 368 μm, an array of letter ‘3’ is successfully recorded by the CCD (Fig. 4b). Nonuniform optical aberrations, which usually originates from imperfect lenses^1^, don’t exist in the image array. It should be also emphasized that the images with similar size is uniform in the same observation plane. These results imply that the unit microlenses in the MLA are perfect and have identical focal length and open aperture, thus promising for real imaging systems with miniaturized footprint.

To verify the resolution of the MLA, a standard resolution test target (1951 USAF, Thorlab) consisting of seven groups of standard patterns with different linewidth etched inside Aluminum film is utilized in the experiment (Fig. S6, Supporting Information). We replaced the standard resolution test target after the plate light source and scanned all the patterns in the target with the microscope stage. The results in Fig. 4c show that the patterns in the group 6 can be clearly distinguished. However, the images of the patterns in group 7 become vague starting from row 6 (R3 in Fig. 4d) and afterwards. In order to further analyze the resolution of the MLA, we captured the light field intensity distribution in the region which is labeled by the white dashed rectangles (R1, R2, and R3 in Fig. 4d) and the distribution profiles along the transverse direction (namely *y* axis) were plotted, respectively. It can be estimated that the light intensity of dark lines in box R3 is 60% of that of the brightest spot, which reveals that it cannot be resolved according to Rayleigh criterion^34^. Moreover, it can be seen from box R2 in Fig. 4d that the dip of the profile is ~42% of the maximum, which implies that these three lines with linewidth of 2.46 μm are on the edge of the distinguishing ability of the MLA. Hence, the critical resolution linewidth of the MLA is estimated to be 2.46 μm, and the corresponding resolvable line pair is 203 LP/mm (the resolvable line pair rules are shown in Table S1). The resolution of our sample (equivalent to 10328 ppi, the quantity of resolvable lines in 1 inch) is 34 times better than the state-of-the-art definition of high-resolution display^35,36^, which usually should be beyond 300 ppi (or equivalent to 84.6 μm linewidth), thus making it highly promising for the high-resolution 3D imaging.

**Fig. 5:**
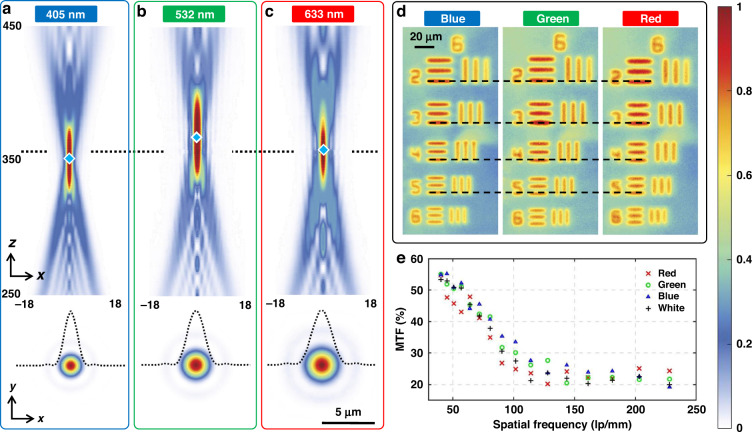
Optical characterization of the chromatism and modulation transfer function of the experimentally produced MLA with diameter of 100 μm. **a**–**c** The focusing property of the MLA under illumination of continuous-wave laser with different working wavelength. The blue rectangle denotes the center of the focal spot along optical axis. **a** *λ* = 405 nm, **b** *λ* = 532 nm, **c** *λ* = 633 nm. **d** The captured images of the samples on the resolution plate (1951 USAF, Thorlab) under illumination of blue, green and red light emitting diodes. **e** The modulation transfer function of the microlens with diameter of 100 μm

In order to comprehensively access the performance of the MLAs with 100 μm diameter, we have performed optical characterizations, including chromatism, full width at half maximum (FWHM), modulation transfer function (MTF), field-of-view (FOV) and focusing efficiency measurement. Figure 5a–c depicts the focusing effect of the MLA under illumination of three continuous-wave lasers with different working wavelengths (405, 532 and 633 nm). The measured focal lengths are given as 350, 366 and 357 μm, respectively. It reveals that the focal lengths at three wavelengths are different, however, the chromatic aberration can be mitigated, due to the small NA and the long depth of focus (DOF) which is proportional to λ/*NA*^*2*^ along the propagation direction. If the images are captured at *z* = 357 μm, the images under different wavelength are still maintained within the range of DOF. The measured FWHM at working wavelengths of 405, 532 and 633 nm are given as 1.36 μm (3.35 λ), 1.8 μm (3.37 λ) and 2.12 μm (3.34 λ), respectively. The chromatism of the MLAs can also be shown by Fig. 5d, which plots the images of the resolution plate under blue, green and red light illuminations at the same position along the propagation direction. The three color images are almost the same to each other, except that the green image is slightly larger than the blue and red images.

Figure 5e depicts the MTFs of the microlens with 100 μm diameter under different illuminations. We use four kinds of plate LED light sources (namely red, green, blue and white) to image the resolution plate (1951 USAF, Thorlab), and obtain the MTFs as a function of the line pair density. It can be inferred that the MLA maintains an acceptable MTF for the high spatial frequency components larger than 30 lp/mm. The MTF is estimated as ~22% when the spatial frequency is beyond 114 lp/mm. The line pairs can still be differentiated even though at spatial frequency up to 228 lp/mm, thus indicating that the MTF of the MLA is extraordinarily excellent. Moreover, we employ the two-dimensional measuring reticle (GCG-YP907, Daheng Optics) to measure the FOV of the MLAs, which is estimated as 25.8°. We adopt the same definition in the study^37^ to measure the focusing efficiency of the MLA at 405, 532 and 633 nm. The focusing efficiency at these three wavelengths are given as 46.2%, 40.8% and 46.3%, respectively, which is comparable to that of dielectric micro-metalens^4,8^.

## Discussion

We report a mold-free and self-assembly approach for producing MLA with high-quality surface and scalability in this article. Theoretically, the resolution of MLA proposed in this study can be further enhanced by using more liquid dose, although the NA of the MLA has a limit once the modified region is fixed. Hence, the dose of both the liquid volume and the oxygen plasma in the process of modification can be controlled to tune the NA and focal length of the MLA according to practical application.

In this study, patterned surface modification is realized by using oxygen plasma and lead to an array with strong chemical contrast between modified and untreated region. This provides a feasible alternative to tailor the curvature and NA of the MLA by tuning the oxygen plasma intensity. The formation of the MLA with smooth morphology can be attributed to the surface tension and the wettability contrast providing sufficient bonding force and constraint for the curable liquid. As proof of concept, we experimentally fabricated MLA with 100 μm diameter and controlled NA based on blade coating method, which possesses smooth surface roughness and allows for an imaging resolution up to equivalently 10,328 ppi (34 times better than the state-of-the-art definition of the high-resolution display). This study shows a cost-effective roadmap for producing MLA in batch, and may find applications in the fast proliferated integral imaging industry and high-resolution display.

## Materials and methods

### Preparation of MLAs based on selective wetting

Glasses with 0.7 mm thickness are employed as the substrate for all the MLA samples. The substrate is cleaned in 3% alkaline water solution for 10 min and then rinsing in deionization (DI) water for 2–3 min. After that, they are dried off by blowing Inert gas (N_2_) and heated in an UV ozone (T16X16/OES, UVOCS) for 5 min. Figure 2b shows the detailed procedures for fabricating MLAs. First, the fluoropolymer (FP) solution (3 wt% Hyflon AD 40H, Solvay) is spin-coated (Spin coater, KW-4A, Institute of microelectronics of Chinese academy of sciences) on the glass substrate at 430 rpm for 30 s. The FP coated substrate is heated on a hot plate (EH20B, LabTech) for 1 min at 85 °C, then is transferred into a reflow oven (KMOL-2, K-SEN) for 1 h at 185 °C. After that, a positive photoresist (SUN-1170N, Suntific Materials) dissolving in propylene glycol methyl ether acetate (PGMEA) and functioning as a sacrificial layer is spin-coated on the surface of the hydrophobic Hyflon film using a step-by-step spin-coating method. The SUN-1170N is spin-coated at 2000 rpm for 15 s, and then at 5000 rpm for 65 s, which is followed by a pre-baking on a hot plate at 85 °C for 5 min. A masked exposure process (Fig. 2biii) is conducted with 365 nm UV light (URE-2000/35, Institute of optics and electronics, Chinese academy of sciences) under 22 W/cm^2^ illumination intensity for 50 s. A uniform sacrificial layer with the thickness of ~22 ± 1 μm is obtained.

After exposure, the sample coated with positive photoresist is directly placed in 1 wt% KOH solution (solvent is pure water) for development for 1.5 min (temperature: 23 °C), and then cleaned with pure water for 1 min (at 23 °C). After that, a set of micro-circular hole-array is shaped (Fig. 2biv). With the mask protection provided by the patterned sacrificial layer, O_2_/plasma based surface modification is conducted in a reactive ion etching (RIE) machine (ME-6A, Institute of Microelectronics of Chinese Academy of Sciences) at 5 W for 10 s (Fig. 2bv). The sacrificial layer is removed by another developing step in PGMEA solution, and finally the hydrophilic/hydrophobic patterned surfaces are obtained (Fig. 2bvi). Under the control of selective wetting behavior, a set of photocurable micro-droplet array (NOA-73, Aladdin) exactly defined by the hole-array is distributed on the substrate by using a blade coating process (MSK-AFA-PD200, Shengzhen Kejing Star Technology Company) at a speed of 0.5 mm/s (Fig. 2bvii). Finally, the MLA with fixed morphology can be get by a 10 s UV curing (Fig. 2bviii).

## Supplementary information

Supplementary materials

Movie 1

Movie 2

## Data Availability

The data that support this study are available from the corresponding authors upon reasonable request.
