# Supplementary material for: Mold-free self-assembled scalable microlens arrays with ultrasmooth surface and record-high resolution
Source: Light Sci Appl. 2023 Jun 7;12:143. doi: 10.1038/s41377-023-01174-7 (PMC10247731; doi:10.1038/s41377-023-01174-7)
Supplement: Supplementary file 1 — Supplementary materials [file 41377_2023_1174_MOESM1_ESM.docx]

**Supplementary Information for**

**Mold-Free Self-Assembled Scalable Microlens Arrays with Ultrasmooth Surface and Record-high Resolution**

Zhihao Liu^1,2^, Guangwei Hu^3^, Huapeng Ye^1,2,*^, Miaoyang Wei^1,2^, Zhenghao Guo^1,2^, Kexu Chen^1,2^, Chen Liu^1,2^, Biao Tang^1,2,*^ and Guofu Zhou^1,2,4,*^

^1^Guangdong Provincial Key Laboratory of Optical Information Materials and Technology & Institute of Electronic Paper Displays, South China Academy of Advanced Optoelectronics, South China Normal University, Guangzhou 510006, China

^2^National Center for International Research on Green Optoelectronics, South China Normal University, Guangzhou 510006, China

^3^School of Electrical and Electronic Engineering, 50 Nanyang Avenue, Nanyang Technological University, Singapore 639798, Singapore

^4^Shenzhen Guohua Optoelectronics Tech. Co. Ltd, Shenzhen 518110, China

**Corresponding authors*: yehp@m.edu.cn; tangbiao@scnu.edu.cn; guofu.zhou@m.scnu.edu.cn

**1. Ellipsometer measurement of Hyflon material**

In our experiment, Hyflon is adopted as hydrophobic material on the glass substrate. The refractive index and extinction coefficient of Hyflon in the visible and near infrared range are experimentally characterized by Ellipsometer (RC2-XI, J. A. Woollam). The red line in Fig. S1 depicts the extinction coefficient of Hyflon, which is close to zero, indicating that the material loss of Hyflon is negligible. The black line in Fig. S1 shows the refractive index of Hyflon, which is approximately 1.33, implying that Hyflon is transparent.


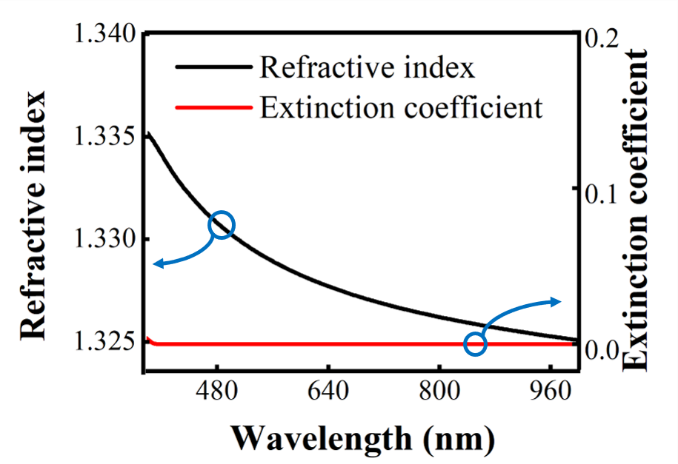


**Figure S1.** Extinction coefficient and refractive index of Hyflon.

**2. Oxygen-plasma surface modification of Hyflon**

The spectrum of the chemical elements consisting of the hydrophobic surface were measured by X-ray photoelectron spectroscopy (XPS). As a fluoropolymer material, its surface hydrophobicity mainly arises from - CF 3 and - CF 2 groups. Hence, the change of C1s element in the oxygen plasma modification was tested in this study.

Figure S2 depicts the XPS spectra (C1s) of Hyflon surface before and after O_2_-plasma modification. It can be seen that the oxygen plasma modification reduces the peak intensity at 293.4 eV while increases the peak intensity near 286 eV. The peak intensity reduction at 293.4 eV (corresponds to the carbon in the -CF3 group) indicates that -CF3 was interrupted on the hydrophobic surface after the modification [1]. The above defluorination process increases the carbon in the polymer structure exposed to the environment, which could explain the peak increase at 286.3 eV. The broken molecular chain could further form hydroxyl (-OH), carboxyl (-COOH), carbonyl (C=O) and other active groups in the highly active oxygen plasma environment [2]. The above surface functional groups may not only enhances the surface hydrophilicity, but also triggers chemical reactions with the photoresist (NOA-73 was applied in our work) at the solid/liquid interface [3, 4]. The binding force caused by the chemical bonds is much stronger than van der Waals force [5], thus leading to better adhesion of NOA-73 microdroplet sitting on the locally modified region.


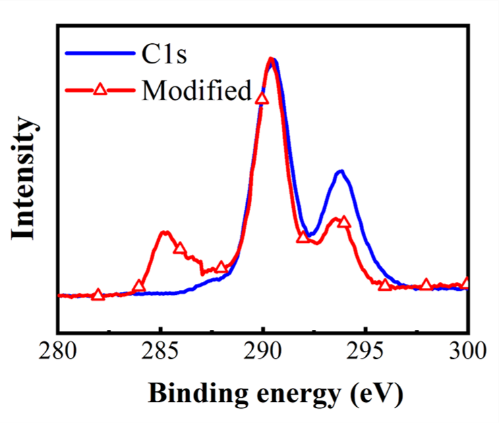


**Figure S2.** XPS spectra (C1s) of Hyflon surface before and after O_2_-plasma modification.

**3. Curvature control of the microdroplet**

The curvature of the microdroplet can be tailored by adjusting the oxygen plasma intensity, which is proportional to the time and power of the plasma in the modification process. The contact angle of water on the modified area changes with the modification time is illustrated in Fig. S3a. When the modification power is kept constant at 5 W, the contact angle (CA) decreases with the increase of modification time. It is evident that the CA changes sharply from 114° to 87.6° when the time is rising up to10s. After that, as the moification time goes on, the change of CA is reletively gentle. This reveals that the critical value of the modification effect is 10s when the modification power is kept constant.

The influence of the modification power has also been studied. In the experiments, we keep the modification time constant at 10s and change the power, as shown in Fig. S3b. The relationship between the wettability of the modified area and the modification power is explored. It can be inferred that the hydrophilicity of the modified area is enhanced with the increase of the power. The CA of the modified area drops sharply from the original 114 ° to 72.1 ° when increasing the power to 80 W. However, the trend of CA becomes gentle when the power is beyond 80 W. Hence, in spite of adjusting the droplet volume, it can be conclude that the modificaion power can also be precisely adjusted to accurately tailor the lens curvature.


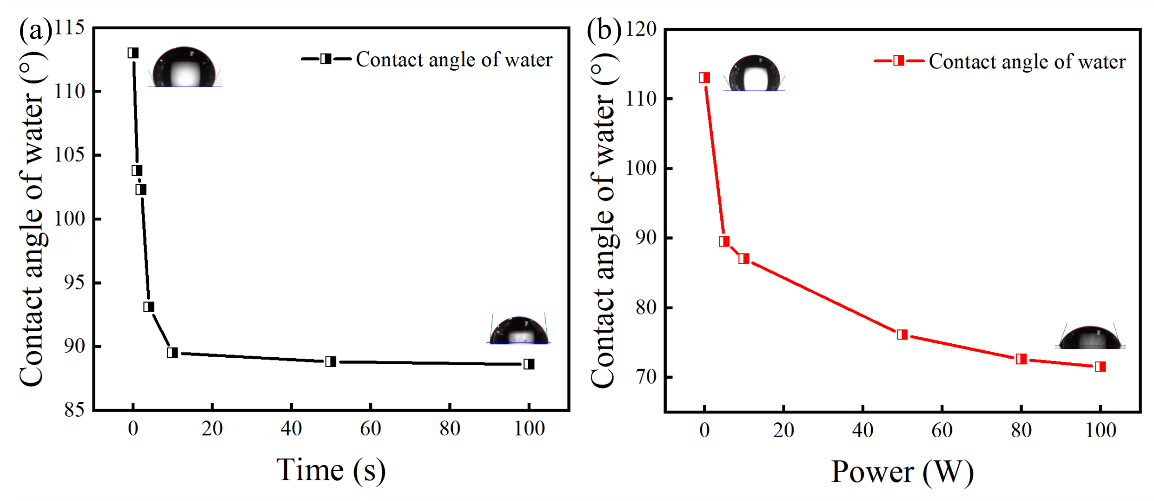


**Figure S****3.** The contact angle of water on the modified area is changed with the modification strength. (a) The surface wettability changes with the modification time, and (b) the modification power.

**4. Morphology and optical performance of MLAs with different aperture**

The microlens arrays (MLAs) with different aperture size are produced in this study. The surface quality of the microlens and the uniformity of the array are judged by its focusing and imaging performance. Figure S4a,b show the microscopic image of the microlens array (MLA) with an aperture size of 100 μm and its corresponding focusing performance at focal length of around 365 μm, respectively. Figure S4c,d are the MLA with an aperture size of 60 μm and the corresponding focal spot at focal length of around 282 μm, respectively. Figure S4e,f are the MLA with an aperture size of 40 μm and the corresponding focal spot at focal length of around 220 μm, respectively. Additionally, the whole focusing process of the MLAs with different aperture can be also seen in Movie 2. These studies help to confirm that the MLAs with different aperture still have good surface quality.

Figure S5 shows the samples with aperture size of 20 μm and 10 μm. It can be seen from the microscopic images that the MLAs have clear and complete lens boundary, which indicates that we can fabricate small size MLAs with higher precision when compared to the MLAs with larger aperture.


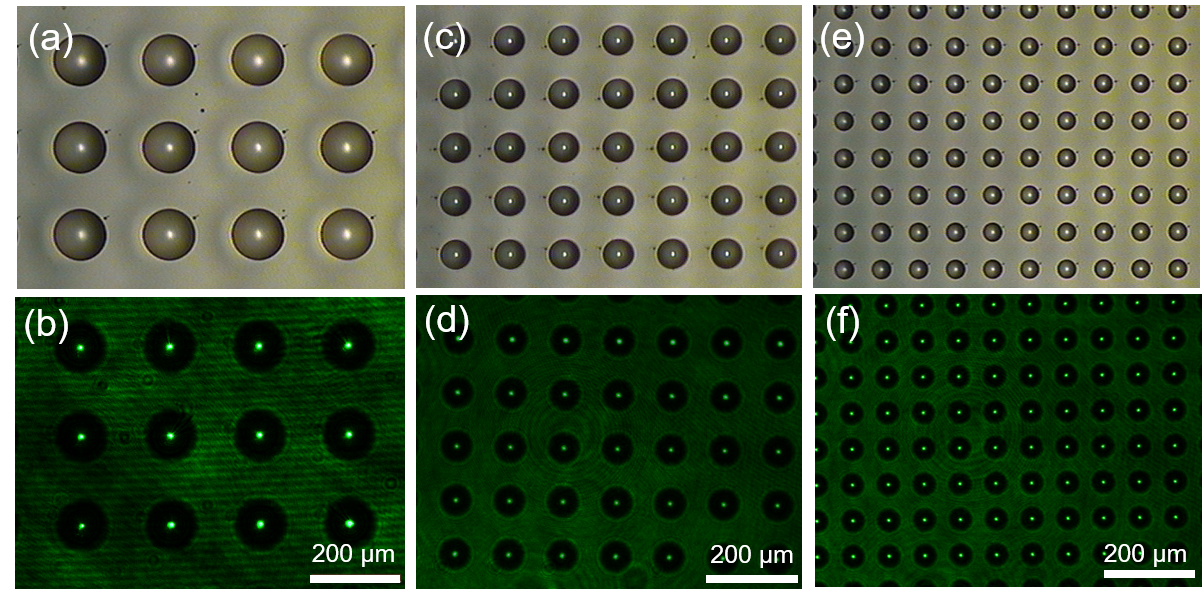


**Figure S4.** Microscopy images of the MLAs samples with different aperture size and its corresponding focal spots. (a) 100 μm and (b) the corresponding focal spots; (c) 60 μm and (d) the corresponding focal spots; (e) 40 μm and (f) the corresponding focal spots.


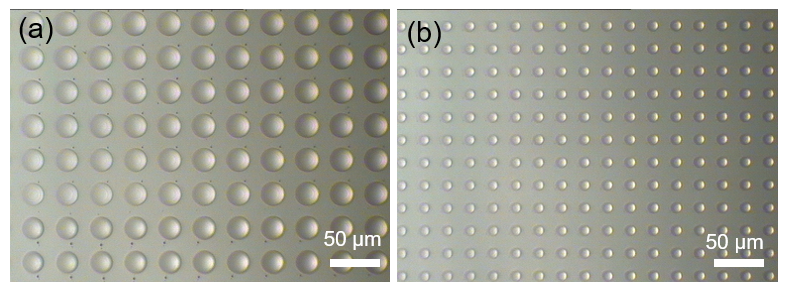


**Figure S5.** Microscopy images of the MLAs samples with aperture size of (a) 20 μm, and (b) 10 μm.

**5. Focusing performance and optical resolution of lens based on selective wetting**

The optical test board (1951 USAF, Thorlab, as shown in Fig. S6) is used to test the resolution of the MLAs. It is consisting of seven groups of patterns with different linewidth, which are marked with different digital labels (The larger the number, the smaller the linewidth). Each group of the pattern contains seven elements. The optical path in the resolution test is similar to the optical path in the imaging. The only difference is that the pattern template "G" in the optical system is replaced by the optical test plate.

It is known from the experiment that the lens can differentiate the fourth element in the fifth group. By comparing the resolvable line pair in Table S1, it is found that the resolvable line pair of the microlens is 203 Lp mm^-1^, and critical resolution linewidth of the microlens is 2.46 μm (equivalent to 10328 ppi).


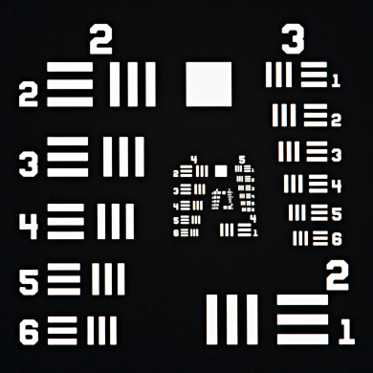


**Figure S6.** Pattern distribution of the optical resolution test board in this study

**Table S1** The resolvable line pair corresponding to the optical resolution test board

| Element Number | Group 1  (Lp mm^-1^) | Group 2 | Group 3 | Group 4 | Group 5 | Group 6 | Group 7  (Lp mm^-1^) |
| --- | --- | --- | --- | --- | --- | --- | --- |
| 1 | 2.00 | 4.00 | 8.00 | 16.00 | 32.00 | 64.00 | 128.00 |
| 2 | 2.24 | 4.49 | 8.98 | 17.95 | 36.00 | 71.80 | 144.00 |
| 3 | 2.52 | 5.04 | 10.10 | 20.16 | 40.30 | 80.60 | 161.00 |
| 4 | 2.83 | 5.66 | 11.30 | 22.62 | 45.30 | 90.50 | 181.00 |
| 5 | 3.17 | 6.35 | 12.70 | 25.39 | 50.80 | 102.00 | 203.00 |
| 6 | 3.56 | 7.13 | 14.30 | 28.50 | 57.00 | 114.00 | 228.00 |

**6. Wettability change of positive photoresist**

We tested the contact angle of water on the surface of positive photoresist (SUN-1170N) to verify the wettability of SUN-1170N. Figure S7a shows that the wettability of SUN-1170N is estimated to be 75.3 ± 1°, showing weak hydrophilicity. Moreover, the contact angle of pure water on hydrophobic layer before modification and after modification is also measured, as shown in Fig. S7b. The contact angle of hydrophobic layer before modification is 114±1°, and becomes 83.8±1° after modification.”

**
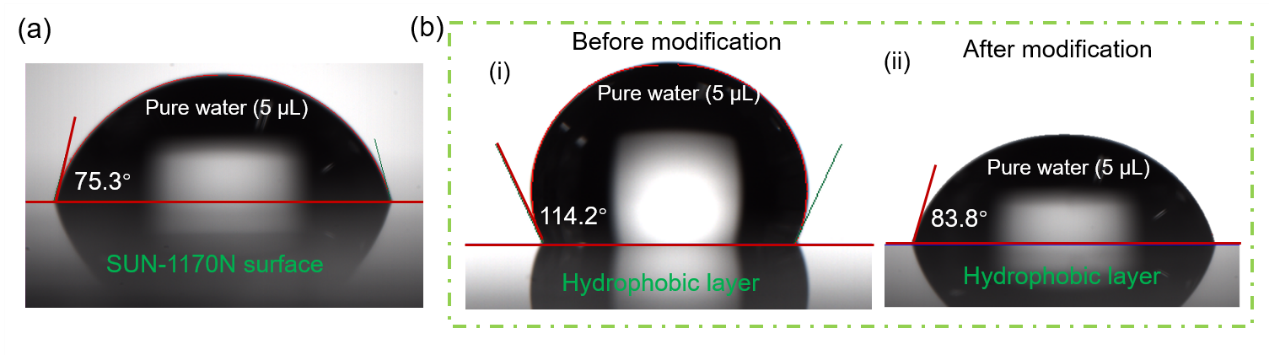
**

**Figure S7**. (a) The wettability of positive photoresist. The contact angle of pure water is 75.3±1°. (b) The wettability of hydrophobic layer (i) before modification and after modification. The contact angle of hydrophobic layer before modification is 114±1°, and becomes 83.8±1° after modification.

**7. Lens size change and the boundary change during the fabrication processing**

We recorded and measured the lens size change and the boundary change during the fabrication processing. Figure S8a shows the exposure area of the patterned template, which is prepared by laser-direct writing technique. The system error in fabrication is ±0.5, and the measured radius of the exposure area is about 50.25 μm. It should be noticed that this template can be recycled. Figure S8b depicts the sacrificial layer area with radius of 50.13 μm, corresponding to the patterned sacrificial layer at Fig. 2b(v) of the article. Figure S8c plots the microlens area after coating and solidification, corresponding to Fig. 2b(viii) of the article.


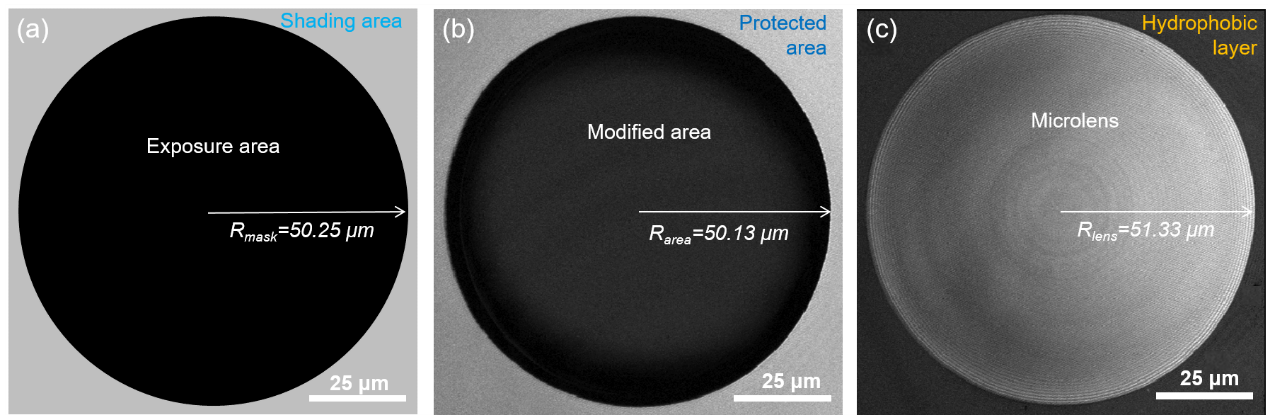


**Figure S8**. The lens size change during the fabrication process. (a) The exposure area on the patterned template (with a radius of about 50.25 μm). (b) Sacrificial layer area prepared with the template (radius=50.13 μm). (c) Microlens area after coating and solidification (radius=51.33 μm).

We captured the images of the exposure area (Fig. S8a), modified area (Fig. S8b) and the lens area (Fig. S8c), respectively. In order to estimate the radii of the circled area in three images, they are analyzed using MATLAB and the binarization method. The radii are calculated with the assistance of the scale bar. The difference between the modified area and the solidified lens radius is approximate 1.2 μm, indicating the radius of the unit size of MLA pattern is well maintained in each step of fabrication process.

**References:**

[1] I. Blakey, G. A. George, D. J. T. Hill, et al., “Mechanism of 157 nm photodegradation of poly (Teflon AF),” Macromolecules 40(25): 8954-8961(2007).

[2] N. Inagaki, S. Tasaka, K. Narushima, et al., “Surface Modification of Tetrafluoroethylene− Perfluoroalkyl Vinyl Ether Copolymer (PFA) by Remote Hydrogen Plasma and Surface Metallization with Electroless Plating of Copper Metal,” Macromolecules 32(25): 8566-8571 (1999).

[3] B. V. Toshev, M. Z. Avramov. “Van der Waals attraction forces and line tension,” Colloids & Surfaces A Physicochemical & Engineering Aspects 100(3): 203-205 (1995).

[4] A. J. Kinloch, A. J. Kinloch, Adhesion and adhesives: science and technology, Springer Science & Business Media, 1987, 58(9): I, III–V, VII–XVI.

[5] A.J. Kinloch, “The science of adhesion,” J. Mater. Sci. 15, 2141-2166 (1980).
